# A New Biomarker of Aging Derived From Electrocardiograms Improves Risk Prediction of Incident Cardiovascular Disease

**DOI:** 10.1016/j.jacadv.2025.101764

**Published:** 2025-05-13

**Authors:** Tom Wilsgaard, Wayne Rosamond, Henrik Schirmer, Haakon Lindekleiv, Zachi I. Attia, Francisco Lopez-Jimenez, David A. Leon, Olena Iakunchykova

**Affiliations:** a Department of Community Medicine, UiT The Arctic University of Norway, Tromsø, Norway; b Department of Epidemiology, University of North Carolina at Chapel Hill, Chapel Hill, North Carolina, USA; c Department of Cardiology, Akershus University Hospital, Lørenskog, Norway; d Institute of Clinical Medicine, Campus Ahus, University of Oslo, Oslo, Norway; e Department of Radiology, University Hospital of North Norway, Tromsø, Norway; f Department of Cardiovascular Medicine, Mayo Clinic College of Medicine, Rochester, Minnesota, USA; g Department of Noncommunicable Disease Epidemiology, London School of Hygiene & Tropical Medicine, London, United Kingdom; h Department of Psychology, University of Oslo, Oslo, Norway

**Keywords:** cohort study, ECG, myocardial infarction, stroke

## Abstract

**Background:**

A biomarker of cardiovascular aging, derived from a deep learning algorithm applied to digitized 12-lead electrocardiograms, has recently been introduced. This biomarker, δ-age, is defined as the difference between predicted electrocardiogram age and chronological age.

**Objectives:**

The purpose of this study was to assess the potential value of δ-age in enhancing the performance of primary prevention models for cardiovascular disease that incorporate traditional cardiovascular risk factors.

**Methods:**

In this cohort study, we included 7,108 men and women from the Norwegian Tromsø Study in 2015 to 16, with follow-up through 2021 for incident fatal and nonfatal myocardial infarction (MI) and hemorrhagic or cerebral stroke. We used Cox proportional hazards regression models, Harrell's concordance statistic (C-index), and the net reclassification improvement.

**Results:**

During a median follow-up of 5.9 years, we observed 155 cases of MI and 141 strokes. In men and women combined,HR per SD increment in δ-age, after adjustment for traditional risk factors included in the Norwegian risk model for acute cerebral stroke and myocardial infarction (NORRISK 2) score, was 1.24 (95% CI: 1.09-1.41) for the combined outcome, with similar HRs for MI and stroke. In men, the HR was significant for MI and in women for stroke. The C-index increased significantly but modestly when δ-age was added to a model with traditional risk factors. The net reclassification improvement was 26.0% (95% CI: 13.3%-38.1%) for the combined outcome, 17.5% (95% CI: 0.6%-33.5%) for MI, and 37.2% (95% CI: 20.1%-53.0%) for stroke.

**Conclusions:**

Incorporating δ-age into primary prevention risk prediction models significantly improved performance beyond traditional cardiovascular risk factors for the combined outcome and separately for MI and stroke.

Machine learning is increasingly used to address issues in biomedicine, creating new scientific insights, and improving diagnosis, management, and risk prediction. Deep neural networks (DNNs) are a machine learning approach that generates diagnostic or predictive algorithms from raw data in an end-to-end manner, that is, without requiring intermediate steps. The application of DNNs to imaging data is proceeding in parallel in several clinical specialties attempting to improve the prevention, diagnostics, and treatment of diseases. In particular, in cardiovascular medicine, DNNs are being developed to process the signals obtained from echocardiography, magnetic resonance images of the heart, and electrocardiograms (ECGs).^1^, ^2^, ^3^, ^4^, ^5^, ^6^ One of the proposed DNNs was a novel biomarker of cardiovascular aging, based on the difference between age predicted using a deep learning algorithm to digitized 12-lead ECGs and chronological age.^1^^,^^6^ Cardiovascular aging results from multiple underlying molecular and cellular processes and occurs at different pace within individuals due to varying exposure to environmental insults, individual behaviours, and genetic predispositions.^7^ Higher cardiovascular aging, defined as having a predicted ECG age greater than one's chronological age has been associated with higher risk of cardiovascular disease (CVD) and total mortality, compared to having a predicted ECG age lower than the chronological age.^6^^,^^8^, ^9^, ^10^ In principle, a biomarker measuring the difference ECG age minus chronological age (δ-age) holds promise to be used as a component of clinical risk prediction scores. It is widely accepted that risk prediction models of CVD, such as SCORE and others, have limitations for predicting individual risk.^11^ Hence, important treatments and life-style change may not be prescribed in time for some patients. Incorporation of δ-age into clinical risk prediction models for CVD may improve risk prediction for these patients and subsequent primary prevention. The objective of this study is to assess δ-age's potential value to improve performance of primary prevention models for CVD that include traditional cardiovascular risk factors. We will use data from 7,008 participants of the Tromsø Study (wave 7, 2015-16) with collected ECGs, and 6 years of median follow-up. We hypothesize that δ-age will improve the prediction of incident fatal and nonfatal myocardial infarction (MI) and stroke on top of what contemporary CVD prediction tools would do.

## Methods

### Study sample

The Tromsø Study is a population-based, prospective study consisting of 7 surveys, referred to as Tromsø1 to 7, conducted in the municipality of Tromsø, Norway, from 1974 to 2016.^12^ In the most recent wave (Tromsø7, 2015-16), all men and women in the municipality aged 40 years and older were invited to participate, with a participation of 65%, amounting to 21,083 individuals.^13^ A subcohort of 8,346 participants, aged 40 to 84 years, was further invited to a second visit, which included more extensive clinical examinations.

For this analysis, the sample is based on the 7,780 participants from Tromsø7 visit 2 who had standard 12-lead ECGs recorded. We excluded participants who did not consent to medical research (n = 2), had a history of MI or stroke (n = 546), or had incomplete covariate history at baseline (n = 124). Thus, the study included 7,108 men and women for the present analyses. The study was approved by the Regional Committee for Medical and Health Research Ethics, REC North. It has conformed to the principles embodied in the Declaration of Helsinki. All participants provided written informed consent.

### Measurements

Tromsø7 incorporated a standardized protocol with physical examinations and blood sampling.^13^ Information regarding smoking habits, education, medication use, and family history of MI was collected through questionnaire data. Resting 10-second digital 12-lead ECG was obtained using the Schiller device AT104 PC, and the raw ECG signal was stored digitally. Blood pressure was measured using an oscillometric digital automatic device (Dinamap Procare 300, GE Healthcare) by trained personnel. After a 2-minute rest in a sitting position, 3 readings were taken on the upper right arm at 1-minute intervals. The average of the last 2 measurements was used to determine systolic blood pressure in this study. Nonfasting blood samples were analyzed using standard methods at the Department of Laboratory Medicine, University Hospital of Northern Norway. Serum total cholesterol and triglycerides were analyzed within 48 hours by enzymatic colorimetric methods using commercial kits from Roche Diagnostics (CHOD-PAP). Serum high-density lipoprotein (HDL) cholesterol was measured after precipitation of low-density lipoprotein using heparin and manganese chloride. Participants' weight and height were measured in light clothing without shoes using a Jenix DS-102 scale (DongSahn Jenix). Body mass index was calculated as weight in kilograms divided by the square of height in meters (kg/m^2^). Smoking status was determined from the response to the question “Do you/did you smoke daily?” with 3 answer options (never; yes, now; yes, previously). The participants were asked to tick which of the following relatives have or have had heart attack before the age of 60 (father, mother, children, siblings). From this a family history of MI before the age of 60 years was created in 3 levels (0, 1, ≥2 family members). Educational level was obtained with the question “What is the highest level of education you have completed?” with 4 answer options (Primary/partly secondary education up to 10 years of schooling; upper secondary education ≥3 years; tertiary education short: college/university <4 years; tertiary education long: college/university ≥4 years).

### Predicted ECG age

We obtained ECG predicted age based on digital 12-lead ECGs for 7,780 participants from Tromsø7 using a convolutional neural networks algorithm developed by our team previously in the Mayo clinic.^1^^,^^8^ The neural network was trained and validated on a large clinical population from the Mayo Clinic (499,727 in the training set, 275,056 in the testing set). A 10-second rested, standard 12-lead ECG was used to develop the neural network. The network was built using stacked blocks of convolutional, max pooling, and batch normalization. The output of the network was the artificial intelligence (AI)-enabled ECG age prediction as a continuous variable.^1^ The predicted ECG age was corrected to account for bias due to systematically higher predicted age in younger participants and lower predicted age in older participants by using linear regression as follows

$$\left(ECGage_{uncorrected}-{age}_{chronological}\right)=\hat{\beta_{0}}+\hat{\beta_{1}}{age}_{chronological}ECGage={ECGage}_{uncorrected}\;–\;\left(\hat{\beta_{0}}+\hat{\beta_{1}}{age}_{chronological}\right)\text{,}$$

where $ECGage_{uncorrected}$ is the uncorrected predicted ECG age, ${age}_{chronological}$ is the observed chronological age, $\hat{\beta_{0}}$ and $\hat{\beta_{1}}$ are estimated linear regression coefficients, and ECGage is the final bias corrected ECG age. Supplemental Figure 1 shows scatter plots between ECG age and chronological age, before and after the bias correction.

The δ-age was calculated as the difference ECGage minus chronological age, where positive values reflect an older than expected age.

### Outcome

Prevalent and incident fatal and nonfatal MI and stroke events were registered from several sources. Events dated 2014 and earlier were sourced from the local CVD register of the Tromsø Study^14^^,^^15^ to identify those with prevalent diseases, while events from 2015 to 2021 were obtained from national registries of MI, stroke, and causes of death. In the local registry, MIs and strokes were identified through linkage to the diagnosis registries at the University Hospital of North Norway and the National Causes of Death Registry. In this registry, we applied modified World Health Organization (WHO) MONICA/MORGAM criteria for defining prevalent MI^14^ and stroke,^15^ with an independent endpoint committee adjudicated both hospitalized and out-of-hospital events. Event cases were ascertained by reviewing medical records with International Classification of Diseases (ICD)-10 discharge diagnoses of I20 to I25, I46 to I48, I50, I60 to I69, G45, G46, G81, R96, R98, or R99. All Norwegian hospitals are mandated to register patients hospitalized with an acute MI in the Norwegian Myocardial Infarction Register and those with acute stroke in the Norwegian Stroke Register. The inclusion criteria for MI encompass all patients with an ICD-10 diagnosis of I21 or I22 hospitalized within 28 days after symptom onset. For stroke, the inclusion criteria are hospitalized patients with ICD-10 codes I61, I63, or I64. Patients hospitalized with acute stroke following a traumatic head injury, stroke related to intracranial tumors, or ischemic stroke following a subarachnoid hemorrhage are excluded from the Stroke Register. The national Cause of Death Registry provided data on out-of-hospital fatal incident cases of MI and stroke. Deaths with ICD-10 codes I21-I24, I25.2, I46, or R96 combined with I20 or I25 as the underlying or contributing cause of death, were defined as MIs. Deaths with ICD-10 codes I61, I63, or I64 were classified as strokes. The diagnoses recorded in the national registers overlapped with the local register for 2013 to 2014 and have been verified as accurate and complete compared with the validated diagnoses in the local register.^16^^,^^17^

### Statistical analysis

All analyses were performed on the whole sample and separately for men and women using SAS software version 9.4 (SAS Institute Inc). Participants with previous MI or stroke at baseline, or incomplete covariate information were not included in the analysis. The analyses were conducted for each outcome, MI and stroke, and for the combined outcomes. Baseline characteristics were presented as means (SD) or percentages (numbers). Follow-up time ranged from the day of study entry in 2015 to 16 to the date of first fatal or nonfatal event, participant censoring due to emigration from Norway, death, or the end of follow-up December 31, 2021, whichever came first. Cox proportional hazard regression models were used to estimate HRs for the association between δ-age and incident fatal and nonfatal events. HRs with 95% CIs were estimated in 2 models: model 1, adjusted for chronological age and sex, and model 2 further adjusted for systolic blood pressure, blood pressure treatment, total cholesterol, triglycerides, HDL cholesterol (dichotomized to <1.00 mmol/L in men and <1.3 mmol/L in women), daily smoking, and a family history of MI before the age of 60 years. The traditional risk factors were selected based on those included the Norwegian risk prediction model NORRISK 2,^18^ which is analogous to SCORE prediction model for primary prevention adapted to the Norwegian population.^18^ Tests of sex differences were performed by adding a two-way cross product between δ-age and sex to a model including main effects of these variables along with all traditional risk factors. Additional Cox models were performed by fitting δ-age with restricted cubic splines with knots at the quintiles and by categorizing δ-age in to 3 levels (ECGage lower than 1 SD (6.2 years) of chronological age, ECG age within ±1 SD of chronological age, ECG age higher than 1 SD of chronological age). Survival curves were estimated using the direct adjustment method with stratified Cox model.^19^ Discrimination was assessed using Harrell's concordance statistic (C-index) and the net reclassification improvement (NRI). Improvement in C-index from the model with the traditional risk factors to the model adding δ-age was estimated as the difference in C-index. The 95% CIs of this difference were estimated from 1,000 bootstrap samples. The NRI was calculated using estimated probabilities of event at 6 years of follow-up from 2 Cox proportional hazard regression models, one with the traditional risk factors and one model with the traditional risk factors and δ-age. We used the version of NRI that is defined as category-free and applicable to survival data.^20^ If we assume n subjects, where n_u_ are reclassified up and n_d_ are reclassified down the NRI can be written as sum of 2 parts as:

$$NRI=\frac{P\left(event|up\right)\cdot n_{u}-P\left(event|down\right)\cdot n_{d}}{n\cdot P\left(event\right)}+\frac{\left(1-P\left(event|down\right)\right)\cdot n_{d}-\left(1-P\left(event|up\right)\right)\cdot n_{u}}{n\cdot \left(1-P\left(event\right)\right)}\text{,}$$

where the numerators represent expected numbers of events reclassified upward and downward (first numerator) and expected numbers of nonevents reclassified downward and upward (second numerator). The denominators are total expected cases of events and nonevents, respectively. The 95% CIs were estimated from 1,000 bootstrap samples. The proportional hazards assumption was verified by visual inspection of log minus log survival curves for quintiles of δ-age and by tests of Schoenfeld residuals.

## Results

We have followed 7,108 men and women from the date of baseline examination in 2015 to 16 to the end of 2021. The median follow-up time was 5.9 years (IQR: 5.4-6.2 years), and we observed 155 incident cases of MI (incidence 3.8 per 1,000 person-years), 141 cases of stroke (incidence 3.4), and 290 cases with either MI or stroke (incidence 7.1). Table 1 shows descriptive characteristics of the study sample. The age at attendance ranged from 39.4 to 85.7 years with an average of 63 years. The baseline predicted ECG age was on average 4.5 years lower than the baseline chronological age, but the average bias corrected predicted ECG age was equal to the average chronological age. The averages for body mass index, systolic blood pressure, total cholesterol, HDL cholesterol, and triglycerides were 27 kg/m^2^, 133 mm Hg, 5.6 mmol/L, 1.6 mmol/L, and 1.5 mmol/L, respectively. Thirty-one percent reported to use blood pressure–lowering medication, 18% used lipid-lowering drugs, 13% were daily smokers, and 44% had reported to have university-level education.

**Table 1:** Descriptive Characteristics by Sex

	Overall (N = 7,108)	Men (n = 3,089)	Women (n = 4,019)
Age, y	63.2 ± 10.4	63.0 ± 10.4	63.3 ± 10.4
Predicted ECG age, y	58.7 ± 8.9	58.4 ± 8.8	58.9 ± 9.0
Bias corrected predicted ECG age, y	63.2 ± 12.1	62.8 ± 11.9	63.4 ± 12.2
δ-age, y	0.00 ± 6.2	−0.17 ± 6.2	0.14 ± 6.1
Body mass index, kg/m^2^	27.2 ± 4.4	27.8 ± 3.9	26.8 ± 4.7
Systolic blood pressure, mm Hg	133 ± 20.2	135 ± 18.1	132 ± 21.5
Blood pressure treatment, %	30.8 (2,186)	32.4 (1,002)	29.5 (1,184)
Total serum cholesterol, mmol/L	5.6 ± 1.1	5.4 ± 1.0	5.7 ± 1.1
Serum HDL cholesterol, mmol/L	1.6 ± 0.5	1.4 ± 0.4	1.8 ± 0.5
Serum triglycerides, mmol/L	1.5 ± 0.9	1.6 ± 1.0	1.4 ± 0.7
Lipid-lowering drug use, %	17.8 (1,268)	18.0 (557)	17.7 (711)
Daily smoking, %			
Current smoker	12.5 (892)	11.8 (366)	13.1 (526)
Previous smoker	48.0 (3,414)	49.8 (1,538)	46.7 (1,876)
Never smoker	39.4 (2,802)	38.4 (1,185)	40.2 (1,617)
Family history of MI before the age of 60 y, %			
0 family members	76.0 (5,377)	78.4 (2,410)	74.2 (2,967)
1 family member	20.7 (1,463)	18.9 (580)	22.1 (883)
≥2 family members	3.3 (232)	2.7 (83)	3.7 (149)
Education			
≤10 y of schooling	28.4 (1,981)	24.7 (750)	31.3 (1,231)
High school diploma	27.9 (1,948)	30.1 (914)	26.3 (1,034)
College or university <4 y	18.6 (1,297)	21.6 (657)	16.3 (640)
College or university ≥4 y	25.1 (1,750)	23.6 (718)	26.2 (1,032)

Values are mean ± SD or % (n).

δ-age = bias corrected predicted ECG age minus chronological age; ECG = electrocardiogram; HDL = high-density lipoprotein; MI = myocardial infarction.

Supplemental Figure 2 shows scatter plots between δ-age and chronological age with filled red star symbols as markers for event outcomes. We observed that mean baseline age was lower in men with MI compared to women with MI, 67.5 years and 72.4 years, respectively, while there was no sex difference in mean baseline age in participants who experienced a stroke during follow-up, 71.1 years vs 71.3 years, respectively.

**Figure 1:**
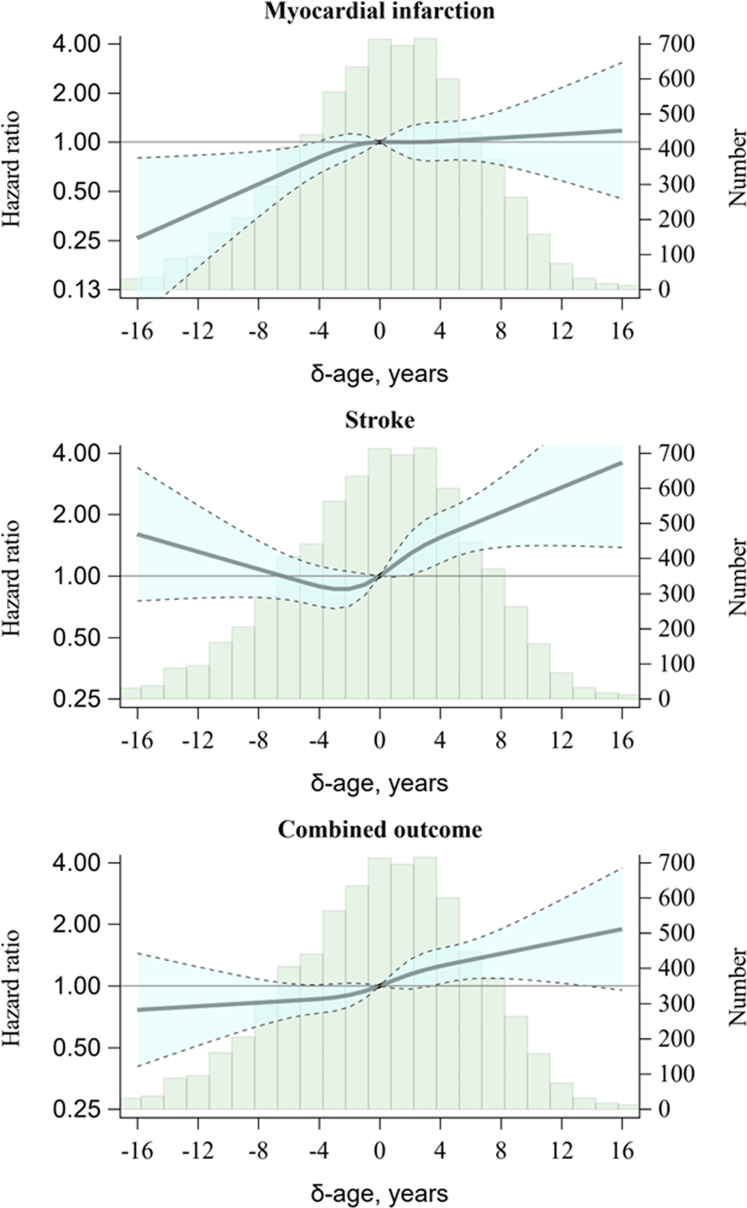
Histograms and HRs for Events According to δ-Age The HRs for the association between δ-age and each outcome are modeled with restricted cubic splines using δ-age equal to zero as reference level. The shaded areas represent 95% confidence limits, and the models are adjusted for age, sex, systolic blood pressure, blood pressure treatment, total cholesterol, triglycerides, low HDL cholesterol, daily smoking, and family history of myocardial infarction before the age of 60 years. δ-age = bias corrected predicted ECG age minus chronological age; HDL = high-density lipoprotein.

HRs per SD increase in δ-age, after adjustment for traditional risk factors, were 1.24 (95% CI: 1.09, 1.41) for the combined outcome, 1.26 (1.06, 1.49) for MI, and 1.25 (1.03, 1.50) for stroke (Table 2). In men, the corresponding HRs were 1.27 (1.09, 1.49), 1.46 (1.18, 1.79), and 1.02 (0.80, 1.31), respectively, and in women, 1.20 (0.97, 1.49), 0.87 (0.64, 1.20), and 1.58 (1.19, 2.11), respectively. Adjustment for the traditional risk factors in the multivariable models only slightly attenuated HR for δ-age compared to models only adjusted for chronological age and sex. We observed significant sex differences in HRs of MI and stroke, but not of the combined outcome. HRs presented in Figure 1, Supplemental Figure 3, and Central Illustration show some slight deviations from the linearity assumption, especially for stroke, but mostly agrees with the results in Table 2 when accounting for the 95% confidence bands. Formal statistical tests showed a significant deviation from linearity in the overall sample with stroke as outcome (*P* = 0.011). Conversely, for the remaining 8 associations, the tests for nonlinearity did not yield significant results, with all *P* values >0.10.

**Table 2:** HRs (95% CI) per SD Increase in δ-Age by Sex

	Combined Outcome	Myocardial Infarction	Cerebral Stroke
Overall			
Number of events	290	156	141
Age- and sex-adjusted model	1.29 (1.14-1.47)	1.32 (1.11-1.56)	1.29 (1.07-1.56)
Multivariable-adjusted model^a^	1.24 (1.09-1.41)	1.26 (1.06-1.49)	1.25 (1.03-1.50)
Men			
Number of events	177	111	68
Age-adjusted model	1.30 (1.11-1.52)	1.49 (1.22-1.83)	1.05 (0.82-1.34)
Multivariable-adjusted model^a^	1.27 (1.09-1.49)	1.46 (1.18-1.79)	1.02 (0.80-1.31)
Women			
Number of events	113	45	73
Age-adjusted model	1.28 (1.04-1.59)	0.96 (0.70-1.31)	1.64 (1.24-2.17)
Multivariable-adjusted model^a^	1.20 (0.97-1.49)	0.87 (0.64-1.20)	1.58 (1.19-2.11)
*P* value for sex equality^b^	0.69	0.007	0.017

Abbreviation as in Table 1.

a HRs are adjusted for age (sex in the overall model), systolic blood pressure, blood pressure treatment, total cholesterol, triglycerides, low HDL cholesterol, daily smoking, and family history of MI before the age of 60 years.

b Test for interaction between sex and δ-age in an overall multivariable adjusted model.

**Central Illustration:**
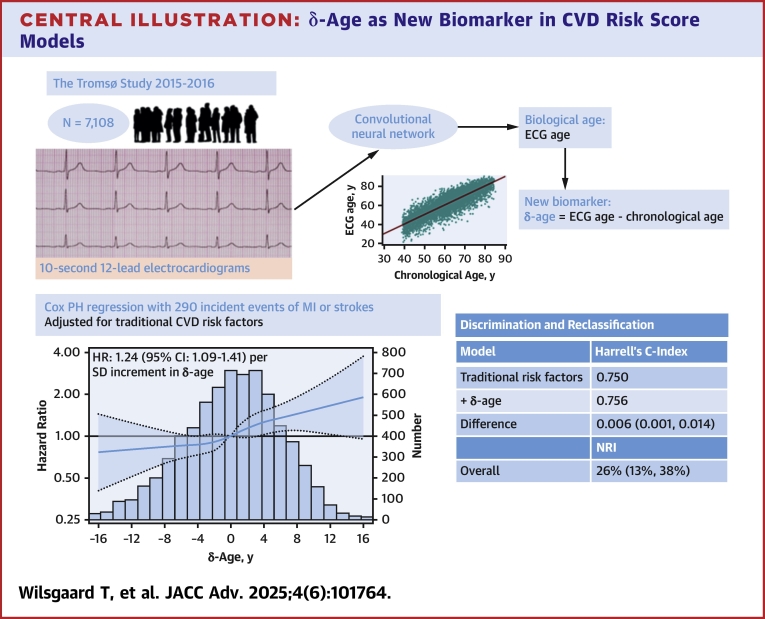
δ-Age as New Biomarker in CVD Risk Score Models Incorporating δ-age into primary prevention risk prediction models significantly improved performance beyond traditional cardiovascular risk factors for myocardial infarction and stroke. CVD = cardiovascular disease; ECG = electrocardiogram; MI = myocardial infarction; NRI = net reclassification improvement; other abbreviation as in Figure 1.

**Figure 2:**
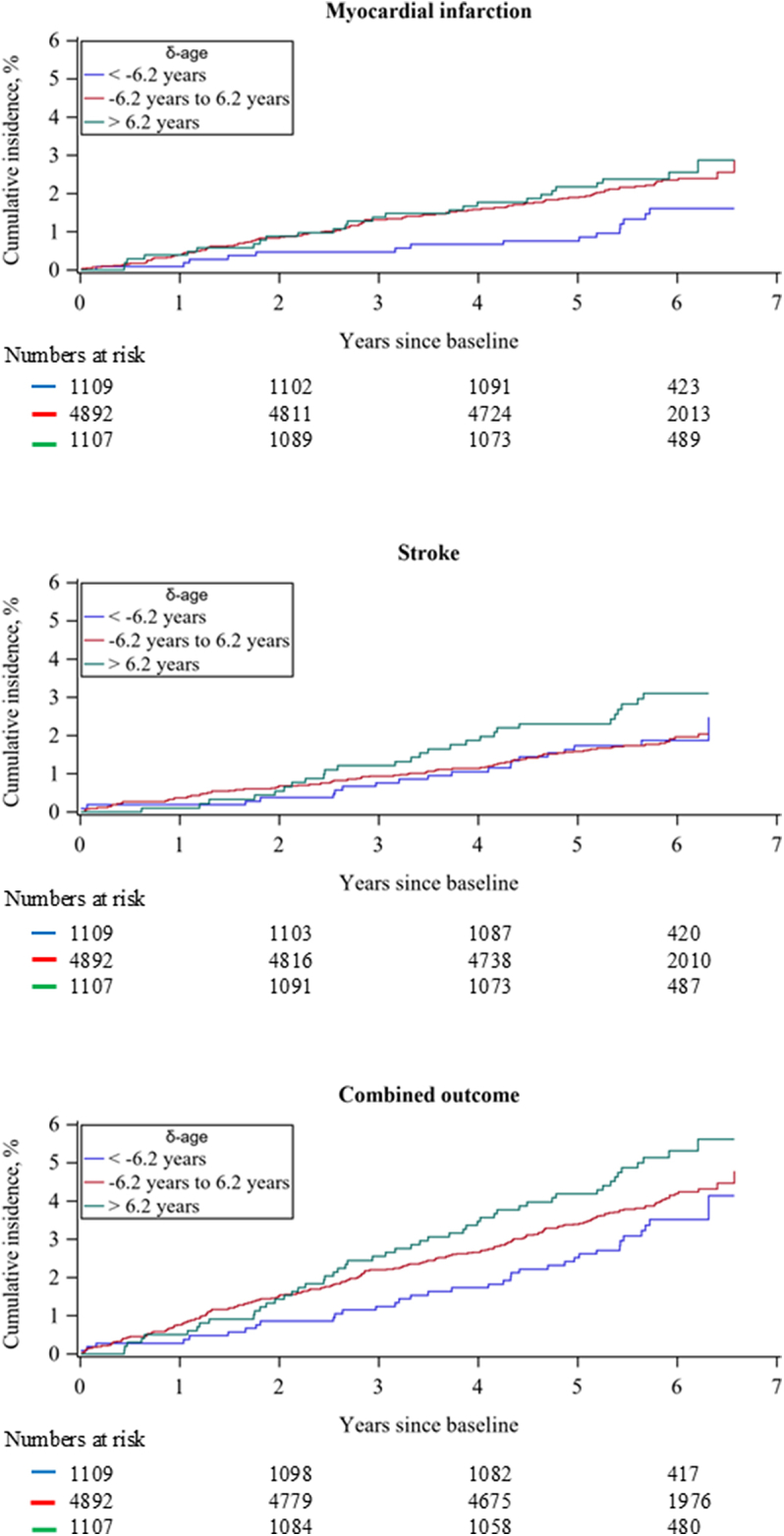
Cumulative Incidence According to δ-Age Levels Cumulative incidence curves are presented for 3 levels of δ-age categorized with cutoffs at ± 1 SD (6.2 years), and estimated from stratified Cox proportional regression models with the direct adjustment method including age, sex, systolic blood pressure, blood pressure treatment, total cholesterol, triglycerides, low HDL cholesterol, daily smoking, and family history of myocardial infarction before the age of 60 years as covariates. Abbreviations as in Figure 1.

A favorable δ-age is a predicted ECG age lower than chronological age, that is, a δ-age <0. The cumulative incidence curves in Figure 2 shows that those with a favorable δ-age below −1 SD have better survival for all outcomes. However, differences between groups were only significant for the combined outcome and borderline significant for MI showing HRs (95% CI) for subjects with δ-age >1 SD compared to δ-age below −1 SD of 1.55 (1.00-2.40) for the combined outcome, 1.80 (0.95-3.43) for MI, and 1.54 (0.85-2.77) for stroke. In sex-specific models (Supplemental Figure 4), these group differences were only significant in men for the combined outcome and MI with HRs (95% CI) of 1.91 (1.10-3.33) and 3.25 (1.44-7.33), respectively.

As shown in Table 3 and Central Illustration, Harrell's C index slightly increased from model 1 with chronological age as the predictor variable to model 2 with the traditional risk factors. A further modest increase was observed between model 2 and model 3 that also included δ-age. The 95% CI for the difference in C-index excluded zero for the combined outcome in both sexes, for MI in men, and stroke in women with differences of 0.006 (95% CI: 0.001-0.014), 0.022 (95% CI: 0.004-0.044), and 0.014 (95% CI: 0.001-0.043), respectively.

**Table 3:** C-Index According to Event by Sex

	C-Index			Difference (95% CI^d^)
	Model 1^a^	Model 2^b^	Model 3^c^	Model 3–Model 2
Overall				
Combined outcome	0.714	0.750	0.756	0.006 (0.001-0.014)
Myocardial infarction	0.734	0.771	0.775	0.004 (−0.000 to 0.012)
Cerebral stroke	0.725	0.755	0.760	0.004 (−0.001 to 0.020)
Men				
Combined outcome	0.663	0.707	0.717	0.010 (0.001-0.024)
Myocardial infarction	0.624	0.690	0.713	0.022 (0.004-0.044)
Cerebral stroke	0.726	0.751	0.751	0.000 (−0.004 to 0.011)
Women				
Combined outcome	0.731	0.776	0.779	0.003 (−0.001 to 0.015)
Myocardial infarction	0.753	0.798	0.800	0.002 (−0.002 to 0.011)
Cerebral stroke	0.728	0.774	0.788	0.014 (0.001-0.043)

a Model 1: Age (and sex in the overall data set).

b Model 2: Model 1 + the traditional cardiovascular disease risk factors systolic blood pressure, blood pressure treatment, total cholesterol, triglycerides, low HDL cholesterol, daily smoking, and family history of MI before the age of 60 years.

c Model 3: Model 2 + δ-age.

d Difference between C-indices with 95% CI from 1,000 bootstrap samples.

Supplemental Tables 1 to 3, Table 4, and Central Illustration show reclassification of events and the NRI statistic for the combined outcome, and separately for MI and stroke, comparing models with traditional risk factors and models including traditional risk factors and δ-age. In both sexes, 17.8% of the expected events for the combined outcome were correctly reclassified up and 8.1% of the expected nonevents were correctly reclassified down resulting in an overall NRI statistic of 26.0% (95% CI: 13.3%-38.1%). The NRIs for MI and stroke were 17.5% (95% CI: 0.6%-33.5%) and 37.2% (95% CI: 20.1%-53.0%), respectively. In sex-specific models, the corresponding NRIs in men were 24.5% (95% CI: 7.7%-40.0%), 19.8% (95% CI: 1.5%-39.6%), and 24.0% (95% CI: −0.0% to 47.8%), respectively, and in women 27.8% (95% CI: 8.4%-46.8%), 1.8% (95% CI: −28.3% to 33.8%), and 51.0% (95% CI: 28.9%-72.4%), respectively.

**Table 4:** Net Reclassification Improvement Indices (95% CI) by Event and Sex^a^

	Combined Outcome	Myocardial Infarction	Cerebral Stroke
Overall			
Among events	17.8% (5.1%-29.7%)	9.8% (−7.1% to 25.2%)	27.7% (10.6%-43.4%)
Among nonevents	8.1% (5.7%-10.6%)	7.7% (5.3%-10.1%)	9.5% (7.1%-12.0%)
Overall	26.0% (13.3%-38.1%)	17.5% (0.6%-33.5%)	37.2% (20.1%-53.0%)
Men			
Among events	15.7% (−0.3% to 30.0%)	7.1% (−11.6% to 26.2%)	20.6% (−2.5% to 43.3%)
Among nonevents	8.8% (5.5%-12.4%)	12.7% (9.3%-16.2%)	3.4% (−0.2% to 7.0%)
Overall	24.5% (7.7%-40.0%)	19.8% (1.5%-39.6%)	24.0% (−0.0% to 47.8%)
Women			
Among events	21.2% (2.0%-39.9%)	−3.3% (−32.9% to 29.4%)	34.4% (12.1%-55.0%)
Among nonevents	6.6% (3.4%-9.8%)	5.2% (2.1%-8.2%)	16.7% (13.6%-19.8%)
Overall	27.8% (8.4%-46.8%)	1.8% (−28.3% to 33.8%)	51.0% (28.9%-72.4%)

95% CI from 1,000 bootstrap samples.

a Net reclassification improvement when δ-age is added to a model with the traditional cardiovascular disease risk factors age, sex, systolic blood pressure, blood pressure treatment, total cholesterol, triglycerides, low HDL cholesterol, daily smoking, and family history of MI before the age of 60 years.

## Discussion

In this population-based study, we have demonstrated that δ-age is independently associated with the combined outcome of MI and stroke, as well as separately for each outcome. The measured effect sizes were only modestly attenuated after adjustment for traditional cardiovascular risk factors. We also showed that incorporating δ-age into cardiovascular risk scores models, such as the Norwegian NORRISK 2, improves predictive accuracy.

A positive value of our biomarker, δ-age, which is the difference AI-generated ECG age minus chronological age, has previously been directly associated with cardiovascular mortality^8^ and major adverse cardiovascular events.^21^ However, those studies were based on primary care patients rather than a general population, and the former did not include nonfatal events. Ladejobi et al^8^ showed that the difference between AI ECG and chronological age is an independent predictor of all-cause and cardiovascular mortality. Their finding aligns with our study, suggesting that the δ-age biomarker could identify individuals aging beyond what is expected from their chronological age. Their study did not present sex-specific results.

Toya et al^21^ included an observational cohort of 531 patients who underwent ECG and peripheral microvascular endothelial function testing at the Mayo Clinic, using the same AI-ECG algorithm as the present study. They found that δ-age was significantly associated with an increased risk of major adverse cardiovascular events in the presence of peripheral microvascular endothelial function, indicating that vascular aging may contribute to cardiovascular risk in people with accelerated physiologic aging.^21^ Their analyses showed a stronger association for women compared to men, although the study was limited by a small number of events. They also demonstrated a significant NRI after adding δ-age to a model including the Reactive Hyperemia Peripheral Arterial Tonometry index.^21^

Another study with a DNN-based ECG-age-prediction model, developed in the Clinical Outcomes in Digital Electrocardiography (CODE) Study cohort,^2^ showed that the difference between predicted ECG age and chronological age is a predictor of overall mortality, despite not being based on an unselected nonpatient population and focusing on overall mortality rather than incident cardiovascular events. This suggests that the difference between predicted ECG age and chronological age could be a useful tool in assessing mortality risk in the general population.

A study from the Tri-Service General Hospital in Taipei developed and validated a deep learning model to estimate ECG age using data from 71,741 first exam ECGs.^3^ They concluded that patients with a high residual (the difference between chronological age and ECG age) had an elevated risk of all-cause mortality, cardiovascular mortality, and cardiovascular outcomes after adjusting for confounding factors. External validation cohorts also demonstrated that a high residual was associated with increased all-cause mortality risk, although the analyses were not sex-specific.

Different AI methods have been applied to predict ECG age and δ-age, and these biomarkers are related to cardiovascular health and total mortality. Biological aging, measured by ECG age, encompasses lifestyle, environmental factors, inheritable and acquired conditions, and diseases.^22^ Our biomarker of biological aging could also relate to other non-CVDs and conditions. For example, another publication from the Tromsø Study showed that δ-age was associated with cognitive function,^23^ indicating potential in identifying individuals at increased risk for neurological conditions.^24^ Also, a large population cohort from the Mayo Clinic demonstrated that social isolation is associated with accelerating biological aging, independent of conventional cardiovascular risk factors.^25^

Our study showed that δ-age was independently associated with the combined outcome of MI and stroke in both men and women. However, in outcome-specific analyses, the associations were only significant for MI in men and for stroke in women, with significant sex differences for both outcomes. Women and men mostly share traditional risk factors for CVD, but the relative impact of these risk factors may vary.^26^ Nevertheless, a common pool of cardiovascular risk factors in men and women contrast our finding that δ-age was not associated with stroke in men and MI in women. Furthermore, several studies have shown associations between specific features derived from standard 12-lead ECGs, like premature ventricular complexes and nonspecific ST-segment and T-wave abnormalities, and coronary heart disease, stroke, and mortality.^27^, ^28^, ^29^, ^30^, ^31^ Some of these studies have presented sex-specific results or tested for sex differences,^27^, ^28^, ^29^ but they do not align with our findings of potential sex difference in the effect of δ-age. Despite observing that women with MI were, on average, 5 years older than their male counterparts and that the statistical power in MI-specific analyses was lower for women than for men, the underlying cause of a possible sex difference in the effect of δ-age remains unclear. This discrepancy warrants further investigation in future population-based studies.

Our study has several strengths. The study cohort is based on a general population with a high attendance proportion. Incident outcome events were extracted from national quality registers for MI and stroke, with all Norwegian hospitals mandated to register patients hospitalized with acute MI and stroke. While the events are not adjudicated, the registers are considered adequately complete and correct.^32^^,^^33^ Furthermore, MI and stroke events from the national registers for 2013 and 2014 were validated against the local adjudicated cardiovascular endpoint register, confirming high correctness and completeness.^16^^,^^17^

### Study limitations

A limitation of the study is that the AI model used to predict ECG age was developed and validated internally using a patient study population where ECGs were obtained for some clinical indication. How the neural network model would perform using a general population is unknown and an external validation in a general population is warranted. However, some key statistics are comparable between the Mayo Clinic and Tromsø Study populations. The mean age is 58.6 years in Mayo Clinic and 63.2 in Tromsø. Pearson correlation coefficient and the mean absolute error between ECG age and chronological age is 0.84 and 0.72, and 6.9 and 6.8 in the Mayo Clinic and Tromsø, respectively. Another limitation, inherent to AI, is the current lack of explainability in deep learning models. This opacity arises not from any specific feature of the image being solely responsible for the outcome, but rather from the totality of information processed. This lack of transparency is met with caution by clinicians. To address this issue, explainable AI aims to elucidate the features of neural networks, thereby making DNNs more acceptable for clinical use. Several randomized control trials are testing or planning to test DNN models in real life situations.^34^, ^35^, ^36^ Understanding human-selected features that AI models are looking at is crucial for adopting this technology in clinical medicine.^37^ For example, Attia et al^37^ demonstrated that neural networks for ECG signals extract features similarly to human experts and generate additional novel features that enhance performance.

## Conclusions

We have demonstrated that δ-age, derived from noninvasive, standard 12-lead ECGs routinely conducted in primary care settings, has significant potential and can be seamlessly integrated into established risk prediction scores, such as the Norwegian NORRISK 2 and the European SCORE model. Our findings should be replicated in other population-based studies with longer follow-up and a greater number of incident events to confirm the robustness and generalizability of our results, and to further elucidate possible sex differences.

Perspectives **COMPETENCY IN MEDICAL KNOWLEDGE:** AI ECG-derived age has the potential to enhance clinical decision-making by playing a significant role in diagnosis, risk stratification, and population-level medicine.

**TRANSLATIONAL OUTLOOK:** Our findings suggest that δ-age could be included in primary prevention risk prediction models for CVD.

## Funding support and author disclosures

The Tromsø Study has been supported by a variety of sources, among them the Northern Norway Regional Health Authority and UiT The Arctic University of Norway. Dr Schirmer has received funding to institution from Novartis as a joint venture research project and lecture fees in person from Amgen, AstraZeneca, Boehringer Ingelheim, Novartis, and Novo Nordisk and has a patent for an algorithm for enhancing detection of valvular heart disease by auscultation. Drs Lopez-Jimenez and Attia are coinventors of the algorithm to determine age using AI-ECG, which algorithm has been licensed to Anumana. The Mayo Clinic and these authors may benefit financially in the future from this licensing. All other authors have reported that they have no relationships relevant to the contents of this paper to disclose.
