# Supplementary material for: A New Biomarker of Aging Derived From Electrocardiograms Improves Risk Prediction of Incident Cardiovascular Disease
Source: JACC Adv. 2025 May 13;4(6):101764. doi: 10.1016/j.jacadv.2025.101764 (PMC12142506; doi:10.1016/j.jacadv.2025.101764)
Supplement: Supplementary data [file mmc1.docx]

**A new biomarker of aging derived from electrocardiograms improves risk prediction of incident cardiovascular disease**

**Supplemental Appendix**

**Table of Contents**

Page 2. Supplemental Figure 1

Page 3. Supplemental Figure 2

Page 4. Supplemental Figure 3

Page 5. Supplemental Figure 4

Page 6. Supplemental Table 1

Page 7. Supplemental Table 2

Page 8. Supplemental Table 3


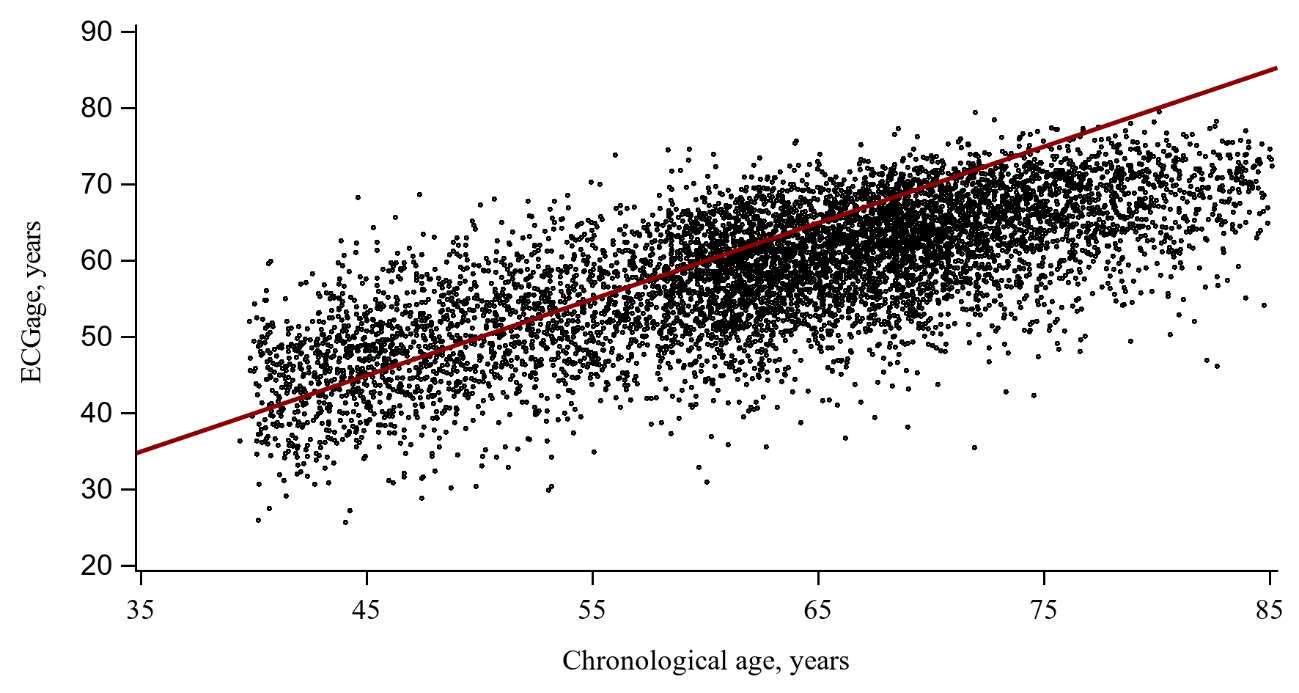


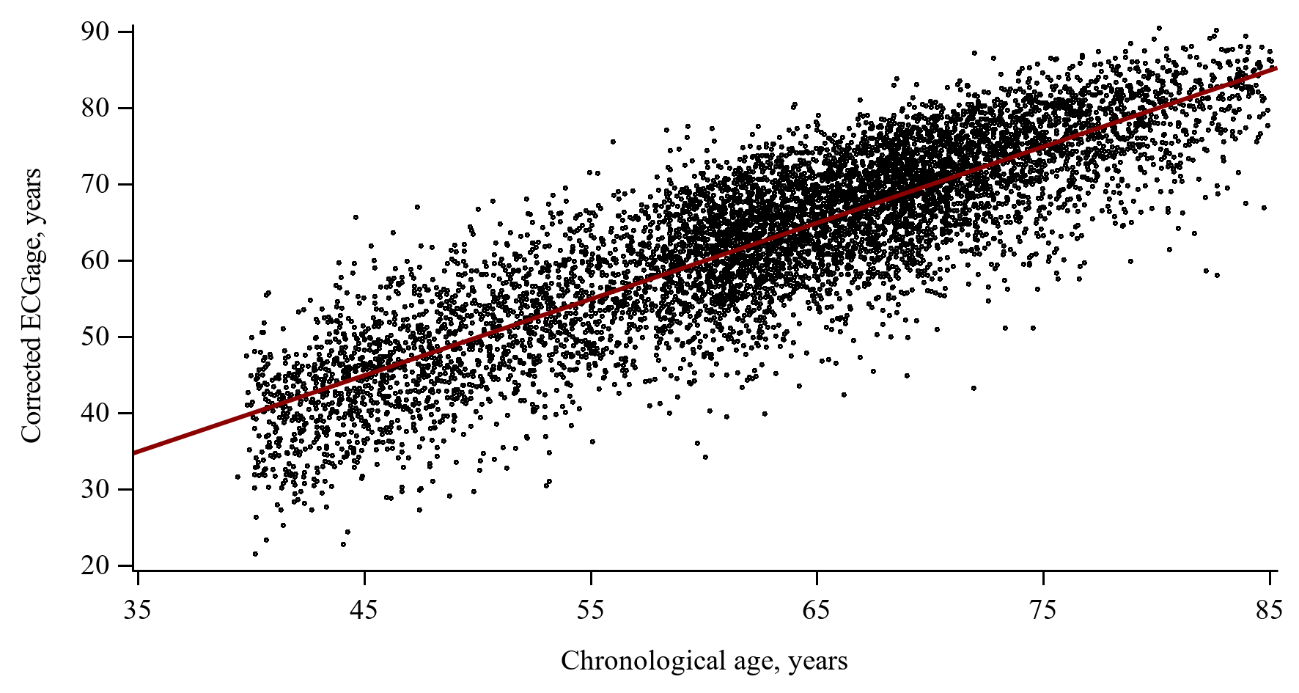


Supplemental Figure 1. Scatter plot between predicted ECG age and chronological age.

The top panel shows scatter plot with the predicted ECG age and the bottom panel show scatter plot with the bias corrected ECG age. The red line id the identity line between ECG age and chronological age.

.


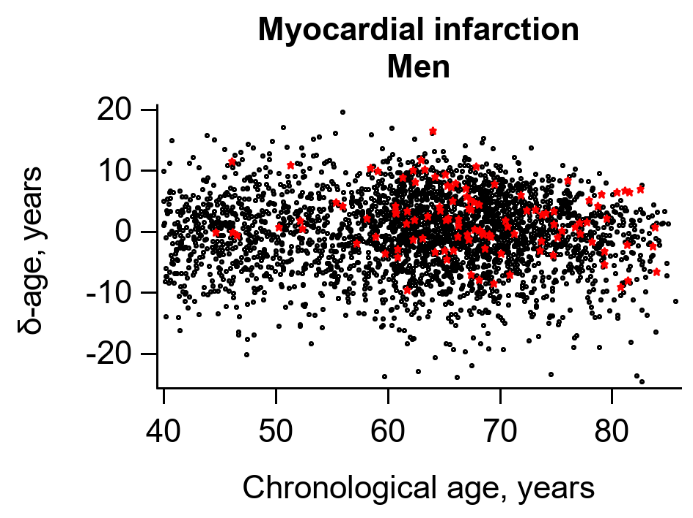

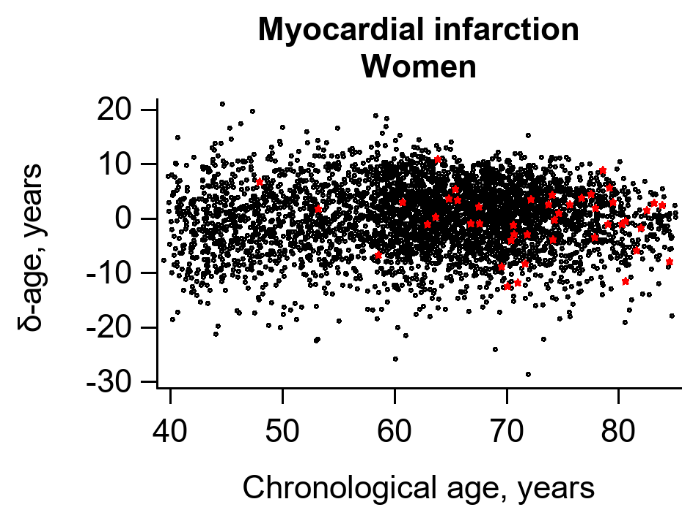


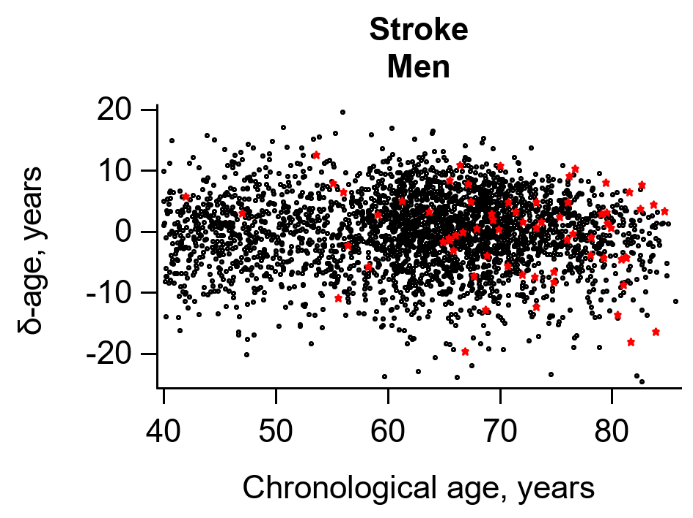

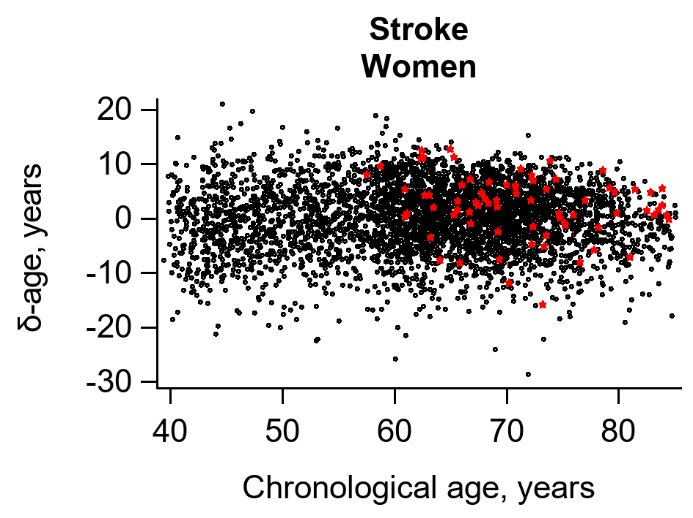


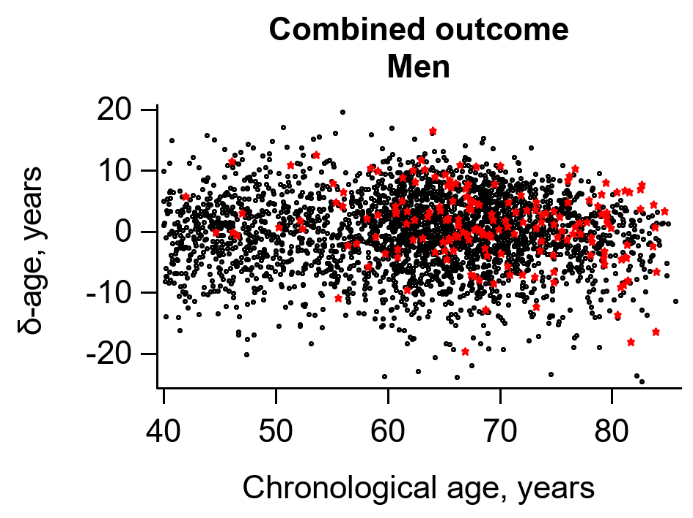

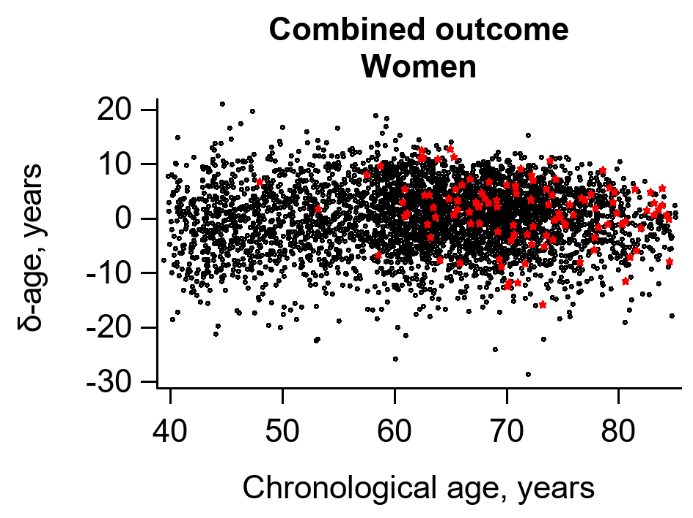


Supplemental Figure 2. Scatter plot between δ-age and chronological age.

The scatter plots are presented according to event type and sex. Event markers are filled red star symbols, while non-event markers are open circles.


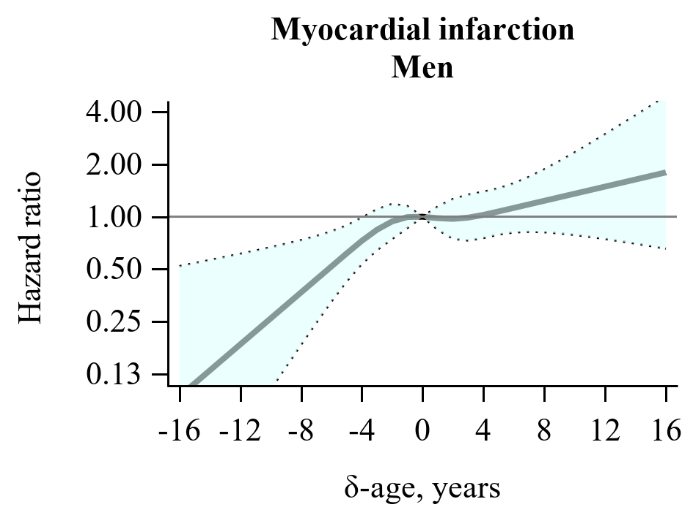

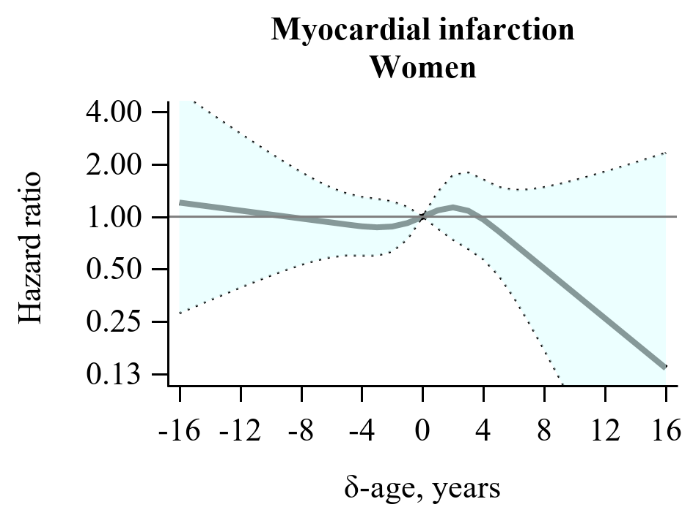


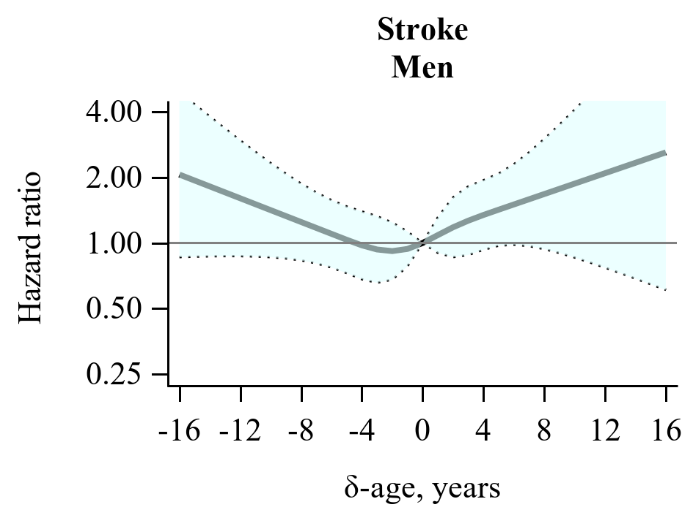

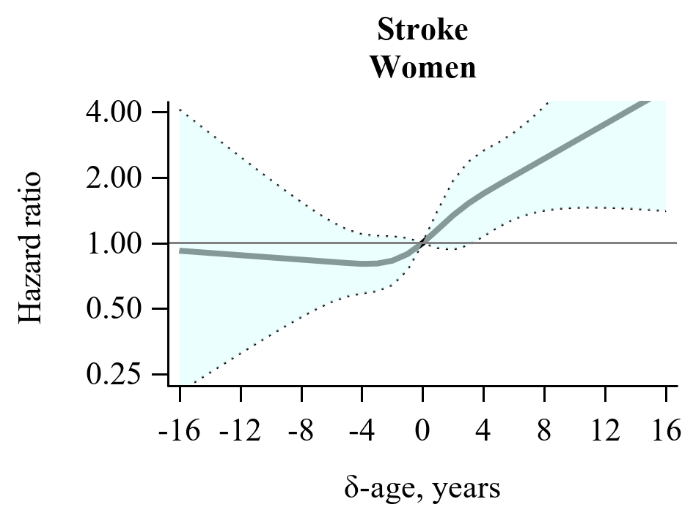


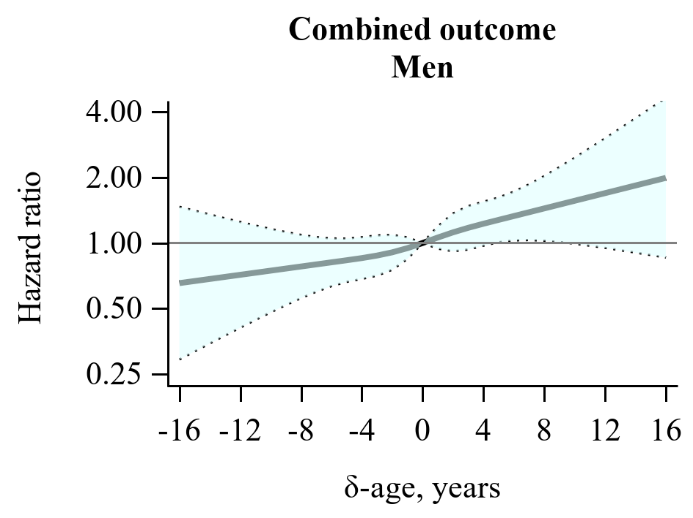

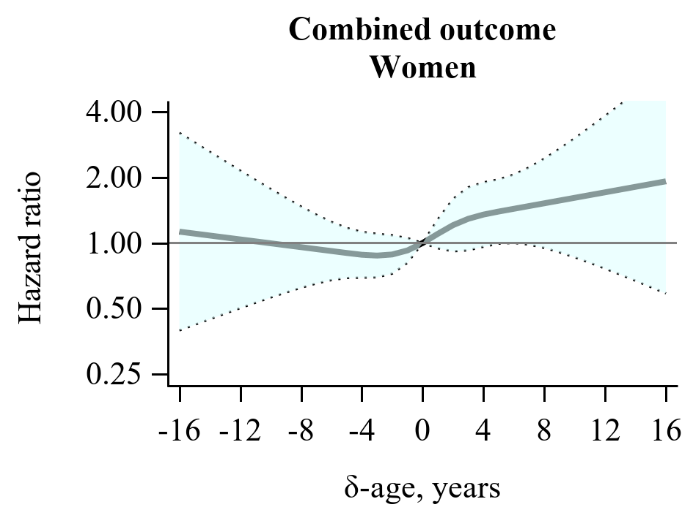


Supplemental Figure 3. Hazard ratios according to δ-age by sex.

The hazard ratios for the association between δ-age and each outcome are modelled with restricted cubic splines using δ-age equal to zero as reference level. The shaded areas represent 95% confidence limits, and the models are adjusted for age, systolic blood pressure, blood pressure treatment, total cholesterol, triglycerides, low HDL cholesterol, daily smoking, and family history of MI before the age of 60 years.


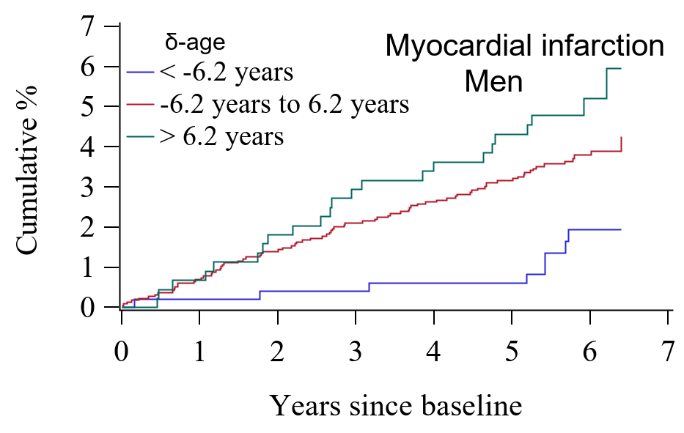

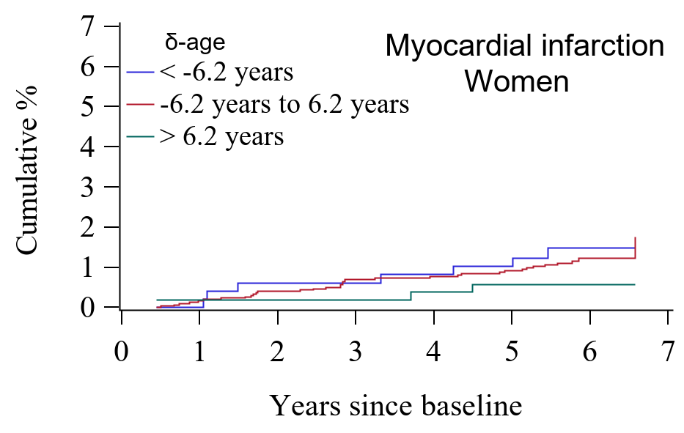


Numbers at risk

499 495 489 199 610 607 602 224

2130 2078 2025 877 2762 2733 2699 1136

460 446 435 197 647 643 638 292


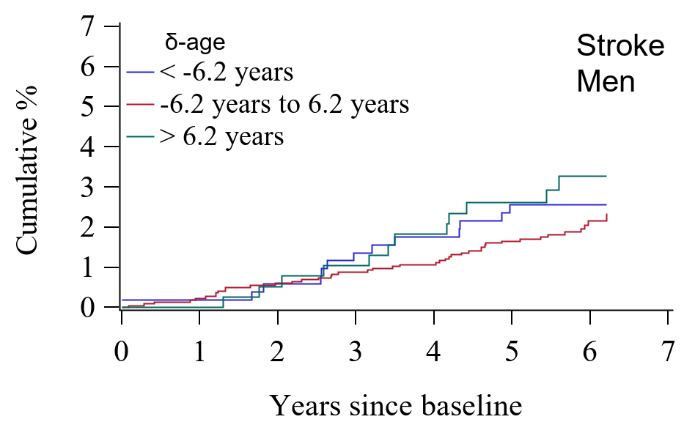

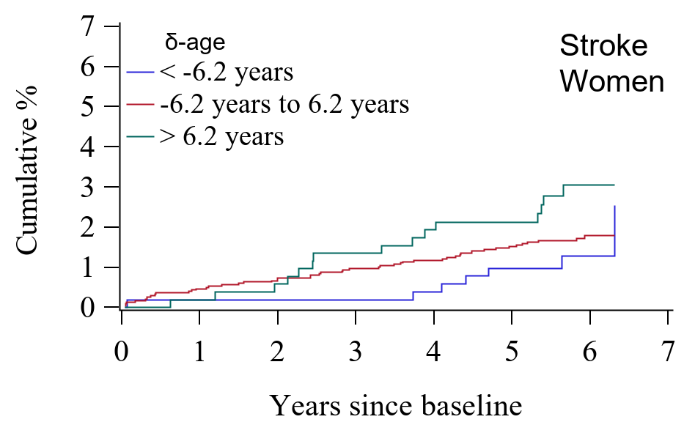


Numbers at risk

499 494 485 197 610 609 602 223

2130 2117 2092 877 2762 2724 2689 1133

460 450 442 202 647 641 631 285


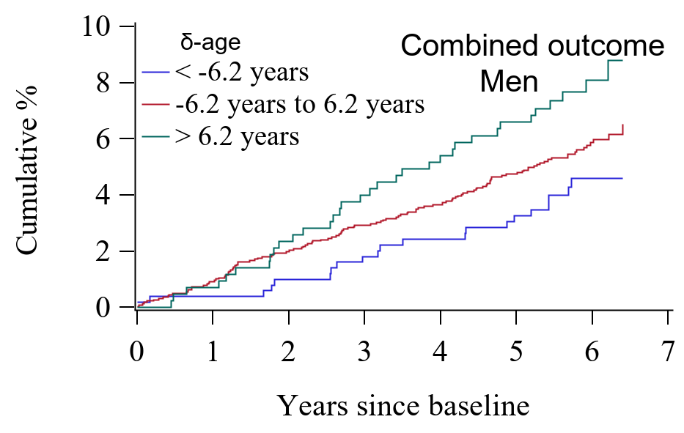

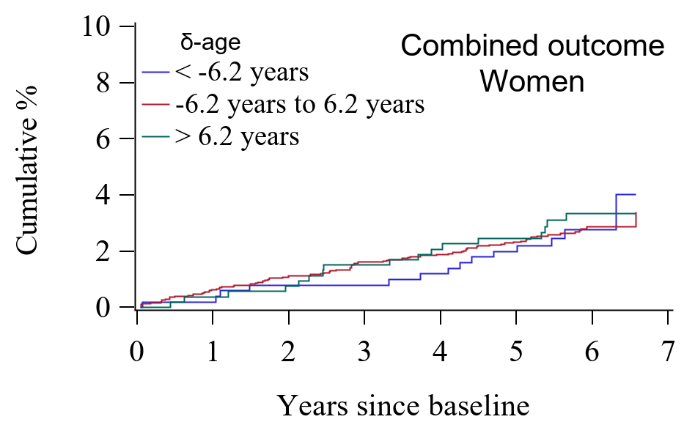


Numbers at risk

499 492 482 195 610 606 600 222

2130 2066 2005 857 2762 2713 2670 1119

460 444 428 195 647 640 630 285

Supplemental Figure 4. Cumulative incidence according to δ-age levels.

Cumulative incidence curves are presented for three levels of δ-age categorized with cut-offs at ± one standard deviation (6.2 years) and estimated from stratified Cox proportional regression models with the direct adjustment method including age, systolic blood pressure, blood pressure treatment, total cholesterol, triglycerides, low HDL cholesterol, daily smoking, and family history of MI before the age of 60 years as covariates.

Supplemental Table 1. Reclassification using predicted probabilities of event as a continuous variable.

|  | All | Reclassified up | Reclassified down |  | NRI (95% CI^a^) |
| --- | --- | --- | --- | --- | --- |
| Combined outcome |  |  |  |  |  |
| Kaplan-Meier rate | 0.0426 | 0.0539 | 0.0327 |  |  |
| Expected number events | 302.7 | 175.2 | 124.3 | Among events | 17.8% (5.1%, 29.7%) |
| Expected number of non-events | 6805.3 | 3125.8 | 3679.7 | Among non-events | 8.1% (5.7%, 10.6%) |
|  |  |  |  | Overall | 26.0% (13.3%, 38.1%) |
| Myocardial infarction |  |  |  |  |  |
| Kaplan-Meier rate | 0.0227 | 0.0269 | 0.0191 |  |  |
| Expected number events | 161.3 | 88.6 | 72.8 | Among events | 9.8% (-7.1%, 25.2%) |
| Expected number of non-events | 6946.7 | 3205.4 | 3741.2 | Among non-events | 7.7% (5.3%, 10.1%) |
|  |  |  |  | Overall | 17.5% (0.6%, 33.5%) |
| Cerebral stroke |  |  |  |  |  |
| Kaplan-Meier rate | 0.0210 | 0.0293 | 0.0139 |  |  |
| Expected number events | 149.1 | 95.1 | 53.8 | Among events | 27.7% (10.6%, 43.4%) |
| Expected number of non-events | 6958.9 | 3147.9 | 3811.2 | Among non-events | 9.5% (7.1%, 12.0%) |
|  |  |  |  | Overall | 37.2% (20.1%, 53.0%) |

^a^95% CI from 1000 bootstrap samples.

CI = confidence interval; MI = myocardial infarction; NRI =net reclassification improvement.

Supplemental Table 2. Reclassification using predicted probabilities of event as a continuous variable in men.

|  | All | Reclassified up | Reclassified down |  | NRI (95% CI^a^) |
| --- | --- | --- | --- | --- | --- |
| Combined outcome |  |  |  |  |  |
| Kaplan-Meier rate | 0.0600 | 0.0748 | 0.0471 |  |  |
| Expected number events | 185.3 | 107.2 | 78.1 | Among events | 15.7% (-0.3%, 30.0%) |
| Expected number of non-events | 2903.7 | 1324.8 | 1578.9 | Among non-events | 8.8% (5.5%, 12.4%) |
|  |  |  |  | Overall | 24.5% (7.7%, 40.0%) |
| Myocardial infarction |  |  |  |  |  |
| Kaplan-Meier rate | 0.0371 | 0.0452 | 0.0308 |  |  |
| Expected number events | 114.7 | 61.4 | 53.3 | Among events | 7.1% (-11.6%, 26.2%) |
| Expected number of non-events | 2974.3 | 1297.6 | 1676.7 | Among non-events | 12.7% (9.3%, 16.2%) |
|  |  |  |  | Overall | 19.8% (1.5%, 39.6%) |
| Cerebral stroke |  |  |  |  |  |
| Kaplan-Meier rate | 0.0237 | 0.0293 | 0.0182 |  |  |
| Expected number events | 73.1 | 44.1 | 29.0 | Among events | 20.6% (-2.5%, 43.3%) |
| Expected number of non-events | 3015.9 | 1456.9 | 1559.0 | Among non-events | 3.4% (-0.2%, 7.0%) |
|  |  |  |  | Overall | 24.0% (-0.0%, 47.8%) |

^a^95% CI from 1000 bootstrap samples.

CI = confidence interval; MI = myocardial infarction; NRI =net reclassification improvement.

Supplemental Table 3. Reclassification using predicted probabilities of event as a continuous variable in women.

|  | All | Reclassified up | Reclassified down |  | NRI (95% CI^a^) |
| --- | --- | --- | --- | --- | --- |
| Combined outcome |  |  |  |  |  |
| Kaplan-Meier rate | 0.0293 | 0.0376 | 0.0218 |  |  |
| Expected number events | 117.6 | 71.2 | 46.3 | Among events | 21.2% (2.0%, 39.9%) |
| Expected number of non-events | 3901.4 | 1822.8 | 2078.7 | Among non-events | 6.6% (3.4%, 9.8%) |
|  |  |  |  | Overall | 27.8% (8.4%, 46.8%) |
| Myocardial infarction |  |  |  |  |  |
| Kaplan-Meier rate | 0.0117 | 0.0119 | 0.0115 |  |  |
| Expected number events | 46.9 | 22.7 | 24.2 | Among events | -3.3% (-32.9%, 29.4%) |
| Expected number of non-events | 3972.1 | 1883.3 | 2088.8 | Among non-events | 5.2% (2.1%, 8.2%) |
|  |  |  |  | Overall | 1.8% (-28.3%, 33.8%) |
| Cerebral stroke |  |  |  |  |  |
| Kaplan-Meier rate | 0.0189 | 0.0301 | 0.0107 |  |  |
| Expected number events | 76.1 | 51.0 | 24.8 | Among events | 34.4% (12.1%, 55.0%) |
| Expected number of non-events | 3942.9 | 1643.0 | 2300.2 | Among non-events | 16.7% (13.6%, 19.8%) |
|  |  |  |  | Overall | 51.0% (28.9%, 72.4%) |

^a^95% CI from 1000 bootstrap samples.

CI = confidence interval; MI = myocardial infarction; NRI =net reclassification improvement.
